# Markers of pulmonary TB in care-seeking patients with respiratory symptoms

**DOI:** 10.5588/pha.24.0034

**Published:** 2025-03-01

**Authors:** A.d.S.R. Moreira, A.P.R. Dalvi, A.L. Bezerra, I.C.d.S. Soares, L.I. Gonçalves, M. Bhering, C.F.d.S. Lara, T.C.P. Dutra, T.d.S.S. Malaquias, E.C. Silva, A.L. Kritski, A.C.C. Carvalho

**Affiliations:** ^1^ Universidade Federal do Rio de Janeiro, Programa Acadêmico de Tuberculose, Faculdade de Medicina, Rio de Janeiro, RJ, Brazil;; ^2^ Universidade do Estado do Rio de Janeiro, Instituto de Medicina Social Hesio Cordeiro, Rio de Janeiro, RJ, Brazil;; ^3^ Fundação Oswaldo Cruz, Instituto Oswaldo Cruz, Laboratório de Inovações em Terapias, Ensino e Bioprodutos, Rio de Janeiro, RJ, Brazil;; ^4^ Fundação Oswaldo Cruz, Escola Nacional de Saúde Pública, Centro de Referência Professor Hélio Fraga, Rio de Janeiro, RJ, Brazil;; ^5^ Departamento Municipal de Duque de Caxias, Centro Municipal de Duque de Caxias, Serviço de Fisiologia, Duque de Caxias, RJ, Brazil;; ^6^ Secretaria de Saúde do Estado do Rio de Janeiro, Fundação de Saúde do Estado do Rio de Janeiro, Rio de Janeiro, RJ, Brazil.

**Keywords:** biomarkers, diagnostic imaging, depression, sociodemographic factors

## Abstract

**SETTING:**

To appropriately triage and evaluate people with signs or symptoms of pulmonary TB, clinical, laboratory, and radiological variables, as well as biomarkers, have been prioritised to increase early detection. However, in high TB prevalence areas, few studies used standardised tools to assess both sociodemographic characteristics and accessible biomarkers comprehensively. This study aimed to describe the sociodemographic, radiographic, clinical, and laboratory characteristics associated with pulmonary TB (PTB) in patients with presumed pulmonary TB (pPTB).

**DESIGN:**

A cross-sectional study was conducted at a public health centre in Duque de Caxias, Brazil, involving patients with pPTB from September 2017 to February 2020. Participants were evaluated using standardised tools: Patient Health Questionnaire 9 (PHQ-9) and the Mini International Neuropsychiatric Interview (MINI) Plus for depression, the MINI-Mental State Examination for cognitive functions, and the ASSIST (Alcohol, Smoking, and Substance Involvement Screening Test) questionnaire for substance use. Chest radiographs (CXRs) and blood tests were also performed. Logistic regression was used to identify associations between sociodemographic, radiographic and biological variables with PTB.

**RESULTS:**

Of 315 patients, 149 (47%) were diagnosed with PTB. Factors associated with PTB included the presence of cavitation on CXR (OR 13.7, 95% CI 5.93–34.5; *P* < 0.001), high alkaline phosphatase levels (OR 3.89; 95% CI 1.68–9.47; *P* = 0.002), and C-reactive protein above 10 mg/L (OR 5.60, 95% CI 2.23–14.7; *P* < 0.001). Major depression disorder (OR 0.33, 95% CI 0.11–0.91; *P* = 0.036) suggested a protective association with PTB.

**CONCLUSION:**

CXR findings and easy-to-perform blood tests can aid in PTB diagnosis, potentially reducing the time to treatment when microbiological or molecular tests cannot be performed.

TB remains a threat to public health and is one of the leading causes of death globally. In 2021, an estimated 10.6 million people fell ill with TB, and 1.6 million died from the disease, reflecting the negative impact of the COVID-19 pandemic on TB elimination actions,^1^ despite the improvement in the adoption of new diagnostic tools and treatment regimens in recent years.^2,3^ The WHO recently reiterated that innovative actions should be included in the TB diagnostic and therapeutic cascade^1^ to detect other prevalent comorbidities, life habits, and catastrophic costs early, which are associated with a higher risk of TB or unfavourable TB treatment outcomes.^4^

In areas with an estimated TB prevalence of 0.5%, the WHO has advocated promoting different screening approaches and the implementation of new interventions, in addition to triage in passive case-finding practices.^2^ These triage interventions should be done in all settings, but it is particularly important to be implemented among people who have higher risk factors for TB. Individuals living in urban poor communities, the homeless, indigenous people, immigrants, people living with HIV, those with diabetes mellitus (DM) or major depression disorder (MDD), alcohol, tobacco or drug users/abusers, household contacts, and inmates are some of the priority groups in which these triage interventions should be implemented.^2,4^ To appropriately triage and evaluate people seeking care who report signs or symptoms of pulmonary TB (PTB), the use of clinical, laboratory, and radiological variables, as well as biomarkers, have been prioritised, alone or in association, to increase the early detection of patients with a higher probability of having PTB.^5–7^ However, in high TB prevalence areas, there is a scarcity of studies that have used standardised and validated instruments to assess sociodemographic factors (including catastrophic costs, comorbidities and life habits) and accessible biomarkers for PTB simultaneously at the front door of health system.^2,4,8^

In this scenario, the present study aims to describe sociodemographic, clinical, radiological and laboratory characteristics associated with PTB in patients with presumed pulmonary TB (pPTB) evaluated in a public health centre with a low prevalence of drug resistance and HIV infection.

## METHODS

### Study design

This is a cross-sectional study carried out from September 2017 to February 2020 involving patients with pPTB at the Municipal Health Center (MHC) of Duque de Caxias (DC). The MHC is in the central region of Duque de Caxias, within the metropolitan area of Rio de Janeiro, RJ, Brazil. It is a secondary-level health facility that functions as a referral centre for TB and HIV, primarily receiving patients with presumed PTB due to the limited decentralisation of TB care to primary healthcare services in the municipality. The population of DC is characterised by a human development index of 0.71, a GINI index of 0.524, and a high proportion of shantytowns. The city is recognised for its high TB burden and low HIV prevalence.^9^

### Participant eligibility and inclusion criteria

Individuals with a cough lasting at least 2 weeks were classified as having presumed pulmonary TB (pPTB). All individuals with pPTB who sought out the health centre in the study period were evaluated for PTB using a clinical score^10^ based only on signs and respiratory symptoms suggestive of TB. Patients aged >18 years and presenting a score >5, with a medium/high probability of PTB,^10^ were invited to participate in the study. PTB was defined as those participants who had positive results in *Mycobacterium tuberculosis* (MTB) culture and/or Xpert^®^ MTB/RIF (Cepheid, Sunnyvale, CA, USA) in sputum samples. None of the patients included in the study presented a disseminated disease.

### Data collection

Participants answered a questionnaire administered by a trained nurse, with questions about sociodemographic, clinical characteristics and life habits. Patients with pPTB underwent anthropometric measurements (weight and height), sputum tests (at least two samples for sputum smear microscopy, Xpert, culture for MTB and first-line drug susceptibility testing (MGIT™ 960™ SIRE; BD, Franklin Lakes, NJ, USA), blood tests (C-reactive protein [CRP], ferritin, leukocytes, haemoglobin, glycated haemoglobin, liver function tests and serology for viral hepatitis and HIV) and chest radiograph (CXR). All blood tests were performed in a certified laboratory (LABORAFE, Rio de Janeiro, RJ, Brazil).

CXRs were evaluated by a pulmonologist, who had no prior knowledge of the patient’s clinical and laboratory data, by using a standardised form that included information on the presence of lung opacities in upper lobes, cavities, and extension of lung involvement (unilateral or bilateral lesions; number of thirds involved). The collection of all clinical, radiological, and laboratory data was performed on patients with probable TB and who had not received drug treatment for TB.

The ASSIST (Alcohol, Smoking, and Substance Involvement Screening Test) questionnaire was used to define tobacco, alcohol, and drug use.^11,12^ A score value between 4 and 15 points is indicative of substance abuse, and values >16 suggest chemical dependence. Body mass index (BMI) was calculated separately for adults and the elderly as recommended by WHO. All cut-offs used in the laboratory variables are presented in the supplementary material (Supplementary Table S1).

The Mini-Mental State Examination (MMSE) was used to evaluate patients’ cognitive functions with the cut-off score according to literacy and age.^13^ The presence of major depressive disorder (MDD) was evaluated first using the Patient Health Questionnaire 9 (PHQ-P9) for screening and the Mini International Neuropsychiatric Interview (MINI) Plus diagnosis. The cut-off score for PHQ-9 used to classify as suggestive of moderate and severe depression was >10 points.^14^ MDD is one of the Mini International Neuropsychiatric Interview (MINI Plus) modules used to confirm diagnosis and can be used by trained mental health specialists, as well as other health professionals.^15^

### Statistical analysis

All demographic, clinical, laboratory and radiographic characteristics and information on costs collected in the baseline were presented in frequencies and proportions. The Fisher exact test and the Wilcoxon rank sum test were used in the primary descriptive analysis. All missing values were excluded from the logistic regression. Bivariate logistic regression analysis was performed to identify associations between PTB and the independent variables. The explanatory variables that presented an association with the outcome (*P* < 0.20) in the bivariate logistic analysis were included in the multivariate Model 1. Variables that presented a high correlation in blood count (red blood cells x haematocrit, neutrophils x lymphocytes), CXR (unilateral or bilateral lesions x number of thirds involved), and hepatitis x liver function tests were excluded from the multivariate analysis. A stepwise multiple logistic regression was conducted in model 1 with a backward selection to identify the characteristics associated with PTB (Final Model). The final model was chosen due to its lower Akaike Information Criterion (AIC). Associations were expressed in terms of odds ratios (OR) and their respective 95% confidence intervals (CI) and *P*-values. The sensitivity, specificity, positive predictive value (PPV), negative predictive value (NPV) and ROC (receiver operating characteristic) curves were used to evaluate the discriminative capacity of the model. All statistical analyses were performed using R Software (R Computing, Vienna, Austria).

### Ethical aspects

This study was conducted according to the principles of the Declaration of Helsinki and approved by the Research Ethics Committee of the University Hospital Clementino Fraga Filho, Rio de Janeiro, RJ, USA, on 2 July 2015, under CAAE number 45637715.5.0000.5257. As required by the Research Ethics Committee, written informed consent was obtained from each study participant.

## RESULTS

Among 315 pPTB patients recruited in the study, 149 (47%) had PTB diagnosis, and 166 (53%) were classified as Non-PTB patients. All the recruited patients were submitted to the clinical score. Sociodemographic and clinical variables more frequently identified among patients with PTB than with Non-PTB were male (67%), age between 18 to 39 years (56%), brown and black skin colour (82%), underweight (35%), no income (55%), and having catastrophic costs (65%). On the other hand, schooling less than 8 years, comorbidities, previous TB treatment and HIV infection were more frequent among patients without PTB. With respect to ASSIST response, tobacco and alcohol use were equally reported by both groups of patients. Drug use was more frequent among patients with PTB (50% vs 39% in Non-PTB) (Supplementary Table S1)

No significant differences were identified between patients with PTB and Non-PTB with respect to moderate to severe cognitive loss or probable moderate to severe MDD. However, current MDD was more frequently reported by patients with Non-TB (22%). On CXRs, cavitation (72%), upper lobe opacities (91%) and bilateral lesions (56%) were more recurrently found among patients with TB (Supplementary Table S2).

The laboratory and biomarkers variables more frequently identified among patients with PTB were prediabetes (46%), anaemia (59%), high neutrophils count (37%), low lymphocytes count (27%), high ferritin level (39%), CRP > 10 mg/L (79%), high alkaline phosphatase (43%), and low albumin level (58%) (Supplementary Table S3). Drug resistance was identified in 17% of the PTB patients. Among these, 11 were resistant to streptomycin, 5 to isoniazid, 1 to ethambutol, 1 to pyrazinamide, and 5 were resistant to both isoniazid and streptomycin.

Among all participants included in the study, 233 individuals (123 Non-TB and 110 PTB) did not present missing values and were included in the logistic regression analysis. The bivariate analysis showed that age (18–39 years), absence of hypertension, no previous TB treatment, lower BMI, need a brief intervention for drug use, cavitation, bilateral or >3 thirds images on CXR, dysglycemia, anaemia, high neutrophil counts, neutrophil/lymphocytes ratio, ferritin, CRP, total bilirubin and alkaline phosphatase levels, and low level of albumin were associated with PTB.

All variables with *P* < 0.20 in bivariate logistic regression analysis and that showed no correlation between them were included in model 1 (Supplementary Table S4). Model 1 identified the presence of cavitation in the CXR, alkaline phosphatase, CRP >10 mg/L, and current MDD episode as variables independently associated with PTB.

In the final model, patients with a current MDD (OR 0.33, 95% CI 0.11–0.91; *P* = 0.036) presented a protective association of PTB when compared with those with an improbable depression diagnosis. The presence of cavitation in the CXR (OR 13.7, 95% CI 5.93–34.5; *P* < 0.001), CRP >10.0 mg/L (OR 5.60, 95% CI 2.23–14.7; *P* < 0.001) and high alkaline phosphatase (OR 3.89, 95% CI 1.68–9.47; *P* = 0.002) were associated with a higher chance of being with PTB (Supplementary Table S4). The accuracy of the model resulted in a sensitivity of 0.83, a specificity of 0.85, a PPV of 0.86 and an NPV of 0.82. The area under the ROC curve was 0.92, indicating the model’s good performance (Figure).

**FIGURE.**
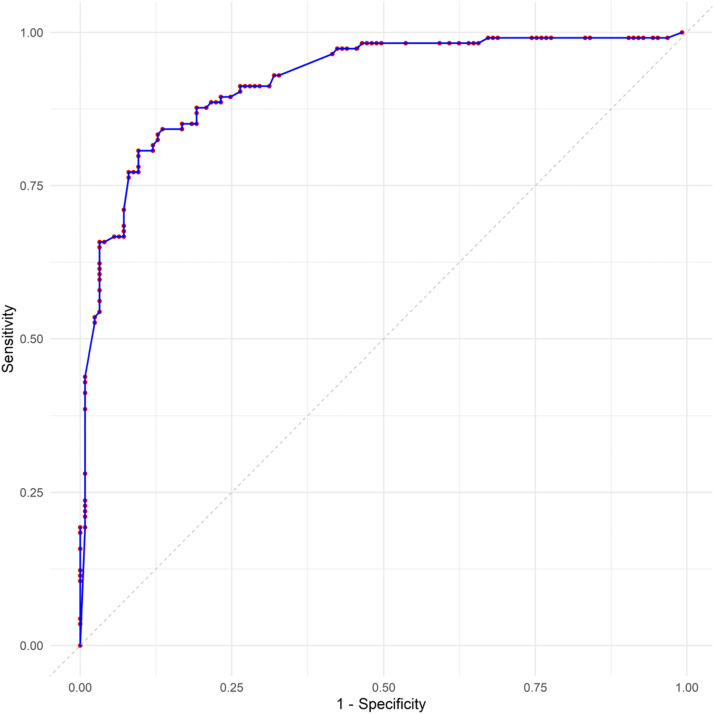
ROC curve showing the distribution of sensitivity and specificity for different cut-off points for the prediction of pulmonary TB. ROC = receiver operating characteristic.

## DISCUSSION

In the present study, we aimed to contribute to the efforts undertaken by the WHO^1–3^ to identify sociodemographic, clinical and radiological conditions, as well as biological markers indicative of a higher probability of PTB. We identified that the presence of cavitation on CXR, alkaline phosphatase, and CRP presented a higher chance of being with PTB, and the current MDD suggested a protective association with PTB.

Patients with PTB were primarily male, aged between 18 and 59 years, non-white and underweight. Lack of monthly income was recurrently reported among patients with PTB. These findings indicate that patients with PTB have worse socio-economic conditions and suffer more from the physical and economic consequences of the disease when compared to patients with other non-TB respiratory conditions, as previously reported in the literature.^2,3,16^

A high proportion of DM and probable MDD, based on PHQ9 response, were found among patients with presumed PTB, as reported previously in regions of high TB burden.^1,17,18^ However, in triage approaches of presumed patients with PTB, data on dysglycaemia and MDD using MINI-Plus are scarce.^2,3,17–19^ We found a high proportion of patients with PTB consuming alcohol, tobacco and drugs, as reported by previous studies.^20–22^ However, most of the studies that evaluated alcohol use among PTB applied the AUDIT (Alcohol Use Disorders Identification Test) instrument^21^ and identified a lower prevalence of alcohol use in patients with PTB^21^ than was identified in our study, but similar to the findings in a previous study in Africa that used the ASSIST^22^ tool. ASSIST is a validated and robust instrument used to confirm the degree of dependence on alcohol, tobacco and drugs and recommended by WHO.^11^

Most studies that evaluated the prevalence of tobacco among patients with PTB used self-reported smoking status,^21^ and its accuracy was recently confirmed by checking the serum cotinine levels.^18^ Studies on TB and drug use are scarce in countries with high TB burden.^22,23^ Drug use in patients with TB was most described among HIV-coinfected patients.^24^ Patients without PTB showed a higher proportion of anti-TB treatment in the past and a lower frequency of prediabetes, but there was no difference in relation to DM between the two groups, as described by others.^17,18^ Among patients with PTB, as we identified in the present study and other high TB burden countries, the prevalence of DM is lower (9% to 21%) than prediabetes (31–33%).^17,25^ The higher frequency of prediabetes compared to diabetes in TB patients may be attributed to the transient nature of hyperglycaemia induced by systemic inflammation during active TB infection.^26^

The level of cognition of patients with pPTB is rarely reported in the literature. We found that a high proportion of patients with pPTB had a low level of cognition, as previously described by Yorke et al.^27^ These findings underline the need for health professionals to be aware and prioritise patients with a lower level of cognition when guiding patient-centred care approach, as proposed by the WHO.^1^ The prevalence of moderate to severe depressive disorder among patients with PTB (45%) was comparable to that described by Duko et al. (39%).^28^ Few studies on TB used MINI-Plus, and in our sample, the prevalence of current and past MDD was lower (20%) than reported by Thungana et al. (38%),^24^ but similar to findings by Alinaitwe et al. (24%).^29^ Interestingly, past MDD occurred more frequently in patients with PTB, while current MDD was more common in the Non-PTB group. Such data suggest that MDD in the past may increase the risk of developing TB, as proposed elsewhere.^30^ This could be influenced by the characteristics of our study sample, which included patients from areas with low socio-economic status and high unemployment, factors that are often linked to mental health alterations.^31^

The WHO recommends using a CXR as a screening tool for pulmonary TB, mainly when bacteriological confirmation is not possible.^32^ In our study, cavitation was strongly associated with PTB, confirming the role this finding may have in PTB diagnostic investigation, as described earlier.^6,33^ Additionally, clinical scores and information and communication technology (ICT) tools can significantly enhance the triage and management of patients with pPTB, particularly in resource-limited settings. This was demonstrated by Libório et al., whose study showed that such approaches reduced the time from triage to obtaining laboratory results and from diagnosis to therapy initiation, which are critical steps in TB management.^34^

Patients with PTB had more frequent CRP levels >10 mg/L, anaemia, elevated ferritin, neutrophilia, high neutrophil/lymphocyte ratio, increased alkaline phosphatase, and lower albumin levels compared with Non-PTB patients, similar to data reported by others.^5,35,36^ However, our findings suggest elevated levels of CRP and alkaline phosphatase, with the presence of cavity on CXR, and lower occurrence of current MDD are associated with PTB. High levels of CRP and cavity on CXRs have been previously identified as a predictor for PTB,^5,6^ but, to the best of our knowledge, not alkaline phosphatase and current MDD. The high level of alkaline phosphatase could be a result of transient liver disease in the initial phase of TB, as proposed by Morris et al.^36^ Related to MDD evaluation, there is scarce data among patients with presumed PTB.^18^

Among the study’s limitations are the high number of variables collected and the limited sample size from a unique health centre, which limits the generalisability of the findings. However, we included eight variables for 110 cases in the final model, resulting in approximately 14 events per variable (EPV), suggesting an unbiased analysis.^37^ The area under the ROC curve (AUC) of 0.92, along with a sensitivity of 0.83, specificity of 0.85, PPV of 0.86 and NPV of 0.82, demonstrates that the model performs well across most decision thresholds, effectively distinguishing between TB-positive and TB-negative cases. Moreover, as a cross-sectional study, causal inferences could not be drawn between variables. On the other hand, the study was carried out by a well-trained team using standardised tools for data collection, and the biochemical tests were performed in a single accredited laboratory.

## CONCLUSION

In summary, our findings may be helpful in decision-making, where CXR findings and easy-to-perform blood tests can reduce the time needed to start TB treatment when other diagnostic methods are not available. The study results further highlight the need to integrate mental health, diabetes and alcohol/tobacco/drug control programmes and social services in managing patients with presumed PTB. There is a need for studies to evaluate whether brief interventions for dysglycemia, MDD, or substance use could improve treatment adherence and outcomes in patients with PTB.

## Supplementary Material


