# Supplementary material for: Markers of pulmonary TB in care-seeking patients with respiratory symptoms
Source: Public Health Action. 2025 Mar 1;15(1):12–6. doi: 10.5588/pha.24.0034 (PMC11841119; doi:10.5588/pha.24.0034)
Supplement: Supplementary file 1 [file pha24-0034_supplementarydata1.pdf]

http://dx.doi.org/10.5588/pha.24.0034

Markers of pulmonary TB in patients with respiratory symptoms searching for care at a health centre

SUPPLEMENTARY DATA

Supplementary Table S1: Laboratorial data cut-offs used for the laboratory variables

		Male	Female
**Red blood cells (millions /mL)**		4.50-6.50	3.90-5.60
**Hemoglobin (g/dL)**		13.5-18.0	11.5-16.4
**Hematocrit (%)**		40-54	36-47
**Neutrophils**		2,500-7,500	
**Lymphocytes**		1,500-3,500	
**Glicade Hemoglobin (HbA1C)**	Normal	Less than 5.7 % Hb Total	
	Risk of Diabetes mellitus	5.7 - 6.4 % Hb Total	
	Diabetes mellitus	More than 6.4 % Hb Total	
**Albumin**		3.5 - 5.5	
**Aspartato Aminotransferase (AST/TGO) (U/L)**		39	37
**Alanina Aminotransferase (ALT/TGP) (U/L)**		45	37
**Alkaline Phosphatase (U/L)**		27 – 100	
**Bilirrubin Total (mg/dL)**		1.0	
**Bilirrubin Direct (mg/dL)**		0.2	
**Bilirrubin Indirect (mg/dL)**		0.6	
**Ferritin**		22.0 - 322.0	10.0 - 291.0

Supplementary Table S2: Demographic, clinical and radiographic characteristics of study participants.

	**Total**[1]	**Non-TB** n= 166	**TB** n= 149	**p-value**[2]
**Sex assigned at birth**				0.064
Female	121 (38.4%)	72 (43.4%)	49 (32.9%)	
Male	194 (61.6%)	94 (56.6%)	100 (67.1%)	
**Age**				<0.001
> 60 years	65 (20.6%)	48 (28.9%)	17 (11.4%)	
18-24 years	46 (14.6%)	16 (9.6%)	30 (20.1%)	
25-39 years	89 (28.3%)	36 (21.7%)	53 (35.8%)	
40-59 years	115 (36.5%)	66 (39.8%)	49 (32.9%)	
**Self-declared skin color**				0.5
White	52 (16.5%)	28 (16.9%)	24 (16.1%)	
Brown	163 (51.8%)	80 (48.2%)	83 (55.7%)	
Black	91 (28.9%)	52 (31.3%)	39 (26.2%)	
Others	9 (2.9%)	6 (3.6%)	3 (2.0%)	
**Marital Status**				0.071
Married	157 (49.8%)	91 (54.8%)	66 (44.3%)	

	Total[1]	Non-TB n= 166	TB n= 149	p-value[2]
Divorced, single or widowed	158 (50.2%)	75 (45.2%)	83 (55.7%)	
**Education (years)**				0.2
More or equal 8 Years	106 (33.8%)	50 (30.3%)	56 (37.6%)	
Less than 8 years	208 (66.2%)	115 (69.7%)	93 (62.4%)	
(Missing)	1	1	0	
**Currently working**				0.5
Working	133 (42.8%)	73 (44.8%)	60 (40.5%)	
Not working	178 (57.2%)	90 (55.2%)	88 (59.5%)	
(Missing)	4	3	1	
**Patient's income**				0.2
Without income	163 (51.8%)	81 (48.8%)	82 (55.0%)	
Less than 1SM	96 (30.5%)	58 (34.9%)	38 (25.5%)	
More than 1SM	56 (17.8%)	27 (16.3%)	29 (19.5%)	
**Government Assistance Program**				0.2
No	267 (84.8%)	145 (87.3%)	122 (81.9%)	
Yes	48 (15.2%)	21 (12.7%)	27 (18.1%)	
**Catastrophic costs**				<0.001
Yes	86 (47.51%)	7 (11.67%)	79 (65.29%)	
No	95 (52.49%)	53 (88.33%)	42 (34.71%)	
(Missing)	134	106	28	
**History of Incarceration**				0.4
No	294 (93.3%)	157 (94.6%)	137 (91.9%)	
Yes	21 (6.7%)	9 (5.4%)	12 (8.1%)	
**Homeless Shelter**				>0.9
No	304 (96.5%)	160 (96.4%)	144 (96.6%)	
Yes	11 (3.5%)	6 (3.6%)	5 (3.4%)	
**Body mass index (BMI)**				<0.001
Underweight	94 (29.8%)	41 (24.7%)	53 (35.6%)	
Healthy Weight	145 (46.0%)	68 (40.9%)	77 (51.7%)	
Overweight	54 (17.1%)	36 (21.7%)	18 (12.1%)	
Obesity	22 (6.9%)	21 (12.7%)	1 (0.7%)	
**Comorbidity**				<0.001
Presence	96 (31.1%)	66 (40.7%)	30 (20.4%)	
Absence	213 (68.9%)	96 (59.3%)	117 (79.6%)	
(Missing)	6	4	2	
**Previous TB treatment**				0.003
No	243 (77.6%)	116 (70.7%)	127 (85.2%)	
Yes	70 (22.4%)	48 (29.3%)	22 (14.8%)	
(Missing)	2	2	0	
**HIV Infection**				>0.9
Positive	24 (7.8%)	13 (7.9%)	11 (7.6%)	
Negative	285 (92.2%)	151 (92.1%)	134 (92.4%)	
(Missing)	6	2	4	

	Total[1]	Non-TB n= 166	TB n= 149	p-value[2]
**Smoking (ASSIST)**				0.3
Never used	82 (29.7%)	39 (26.7%)	43 (33.1%)	
Does not require intervention	79 (28.6%)	47 (32.2%)	32 (24.6%)	
Needs a brief ntervention	99 (35.9%)	54 (36.9%)	45 (34.6%)	
Need more intensive intervention	16 (5.8%)	6 (4.1%)	10 (7.7%)	
(Missing)	39	20	19	
**Alcohol (ASSIST)**				0.5
Never used	40 (14.5%)	20 (13.7%)	20 (15.4%)	
Does not require intervention	109 (39.5%)	64 (43.8%)	45 (34.6%)	
Needs a brief ntervention	114 (41.3%)	56 (38.4%)	58 (44.6%)	
Need more intensive intervention	13 (4.7%)	6 (4.1%)	7 (5.4%)	
(Missing)	39	20	19	
**Drugs (ASSIST)**				0.081
Never used	187 (67.8%)	107 (73.3%)	80 (61.5%)	
Does not require intervention	39 (14.1%)	20 (13.7%)	19 (14.6%)	
Needs a brief ntervention	40 (14.5%)	14 (9.6%)	26 (20.0%)	
Need more intensive intervention	10 (3.6%)	5 (3.4%)	5 (3.9%)	
(Missing)	39	20	19	
**Mini Mental**				0.4
Normal	161 (58.6%)	84 (57.5%)	77 (59.7%)	
Mild cognitive loss	67 (24.4%)	33 (22.6%)	34 (26.4%)	
Moderate and severe cognitive loss	47 (17.1%)	29 (19.9%)	18 (13.9%)	
(Missing)	40	20	20	
**PHQ9**				0.5
No depression	87 (31.5%)	42 (28.8%)	45 (34.6%)	
Mild depressive disorder	52 (18.9%)	n25 (17.1%)	27 (20.8%)	
Moderate depressive disorder	54 (19.6%)	30 (20.6%)	24 (18.5%)	
Moderately severe depressive disorder	41 (14.9%)	22 (15.1%)	19 (14.6%)	
Severe depressive disorder	42 (15.2%)	27 (18.5%)	15 (11.5%)	
(Missing)	39	20	19	
**Mini Plus**				0.035
Improbable depression diagnosis	212 (76.8%)	108 (73.9%)	104 (80.0%)	
Current major depressive episode	49 (17.8%)	33 (22.6%)	16 (12.3%)	
Past major depressive episode	15 (5.4%)	5 (3.4%)	10 (7.7%)	
(Missing)	39	20	19	
**Cavitation on chest radiograph**				<0.001
Presence	125 (39.9%)	18 (10.9%)	107 (72.3%)	
Absence	188 (60.1%)	147 (89.1%)	41 (27.7%)	
(Missing)	2	1	1	
**Upper lobe oppacities**				<0.001
Absent	106 (34.2%)	93 (57.1%)	13 (8.8%)	
Present	204 (65.8%)	70 (42.9%)	134 (91.2%)	
(Missing)	5	3	2	

	Total[1]	Non-TB n= 166	TB n= 149	p-value[2]
**Unilateral or bilateral lesion on chest radiograph**				<0.001
Absent	85 (27.1%)	80 (48.5%)	5 (3.4%)	
Unilateral	103 (32.8%)	43 (26.1%)	60 (40.3%)	
Bilateral	126 (40.1%)	42 (25.5%)	84 (56.4%)	
(Missing)	1	1	0	

[1] n (%); Median (IQR).
[2] Fisher's exact test; Wilcoxon rank sum test.

Supplementary Table S3: Laboratory and biomarkers characteristics of study participants.

	Total[1]	Non-TB n = 166	TB n = 149	p-value[2]
**Drug Sensitivity testing**				
Resistant	23 (7.3%)	0 (0.0%)	23 (17.4%)	N.A.
Sensitive	109 (34.6%)	0 (0.0%)	109 (82.6%)	
(Missing)	10	N.A.	10	
**Dysglycemia**				0.004
Absent	153 (48.9%)	94 (56.6%)	59 (40.1%)	
Diabetes	44 (14.1%)	24 (14.5%)	20 (13.6%)	
Prediabetes	116 (37.1%)	48 (28.9%)	68 (46.3%)	
(Missing)	2	0	2	
**Anemia**				<0.001
Absent	183 (58.5%)	122 (73.5%)	61 (41.5%)	
Present	130 (41.5%)	44 (26.5%)	86 (58.5%)	
(Missing)	2	0	2	
**Hematocrit**				0.091
Normal	209 (67.2%)	118 (71.5%)	91 (62.3%)	
Low	102 (32.8%)	47 (28.5%)	55 (37.7%)	
(Missing)	4	1	3	
**Neutrophils**				<0.001
Normal	196 (63.2%)	108 (65.5%)	88 (60.7%)	
Low	31 (10.0%)	28 (16.9%)	3 (2.1%)	
High	83 (26.8%)	29 (17.6%)	54 (37.2%)	
(Missing)	5	1	4	
**Lymphocytes**				0.006
Normal	233 (74.9%)	129 (78.2%)	104 (71.2%)	
Low	66 (21.2%)	26 (15.8%)	40 (27.4%)	
High	12 (3.9%)	10 (6.1%)	2 (1.4%)	
(Missing)	4	1	3	
**Inflammatory Marker Neutrophils/Lymphocytes**				<0.001
Median (IQR)	3.19 (1.9, 4.6)	2.1 (1.5, 3.5)	4.2 (3.1, 5.1)	

	Total[1]	Non-TB n = 166	TB n = 149	p-value[2]
(Missing)	5	1	4	
**Ferritin**				<0.001
Normal	201 (72.1%)	121 (81.8%)	80 (61.1%)	
High	78 (27.9%)	27 (18.2%)	51 (38.9%)	
(Missing)	36	18	18	
**Albumin**				<0.001
Normal	183 (59.2%)	122 (74.9%)	61 (41.8%)	
Low	126 (40.8%)	41 (25.2%)	85 (58.2%)	
(Missing)	6	3	3	
**AST**[3]				0.9
Normal	268 (85.9%)	141 (85.5%)	127 (86.4%)	
High	44 (14.1%)	24 (14.6%)	20 (13.6%)	
(Missing)	3	1	2	
**ALT**[4]				0.7
Normal	268 (85.9%)	143 (86.7%)	125 (85.1%)	
High	44 (14.1%)	22 (13.3%)	22 (14.9%)	
(Missing)	3	1	2	
**Bilirrubin Total**				0.12
Normal	297 (94.9%)	161 (96.9%)	136 (92.5%)	
High	16 (5.1%)	5 (3.0%)	11 (7.5%)	
(Missing)	2	0	2	
**Bilirrubin Direct**				0.11
Normal	146 (46.8%)	85 (51.2%)	61 (41.8%)	
High	166 (53.2%)	81 (48.8%)	85 (58.2%)	
(Missing)	3	0	3	
**Bilirrubin Indirect**				0.4
Normal	301 (96.5%)	161 (97.6%)	140 (95.2%)	
High	11 (3.5%)	4 (2.4%)	7 (4.8%)	
(Missing)	3	1	2	
**Alkaline phosphatase**				<0.001
Normal	209 (67.2%)	126 (76.4%)	83 (56.9%)	
High	102 (32.8%)	39 (23.6%)	63 (43.2%)	
(Missing)	4	1	3	
**C-reactive protein (mg/L)**				<0.001
<6.0	96 (30.8%)	81 (48.8%)	15 (10.3%)	
6.0 a 10.0	61 (19.6%)	45 (27.1%)	16 (10.9%)	
> 10.0	155 (49.7%)	40 (24.1%)	115 (78.8%)	
(Missing)	3	0	3	
**Anti HBC Total**				>0.9
Positive	32 (11.8%)	17 (11.6%)	15 (12.0%)	
Negative	240 (88.2%)	130 (88.4%)	110 (88.0%)	
(Missing)	43	19	24	
**HBS-Ag**				0.5

	Total[1]	Non-TB n = 166	TB n = 149	p-value[2]
Positive	1 (0.37%)	0 (0.0%)	1 (0.8%)	
Negative	268 (99.3%)	147 (100.0%)	121 (99.2%)	
(Missing)	46	19	27	
**Anti HCV**				0.6
Positive	12 (4.3%)	8 (5.3%)	4 (3.2%)	
Negative	265 (95.7%)	143 (94.7%)	122 (96.8%)	
(Missing)	38	15	23	

[1] n (%); Median (IQR);
[2] Fisher's exact test; Wilcoxon rank sum test; N.A.: not aplicable
[3]AST – Aspartate aminotransferase
[4]ALT – Alanine aminotransferase

Supplementary Table S4: Multivariate logistic regression results of variables associated with pulmonary tuberculosis.

	Model 1 (p-value<0.2)			Final Model		
	**OR[1]**	**95% CI[1]**	**p-value**	**OR[1]**	**95% CI[1]**	**p-value**
**Sex**						
Female	—	—				
Male	1.18	0.42, 3.37	0.75			
**Age**						
> 60 years	—	—				
18-24 years	3.67	0.63, 23.3	0.16			
25-39 years	2.70	0.63, 12.2	0.19			
40-59 years	1.72	0.46, 6.69	0.42			
**Marital Status**						
Married	—	—				
Divorced, single or widowed	0.79	0.30, 2.01	0.62			
**Education (years)**						
More or equal 8 Years	—	—		—	—	
Less than 8 years	0.63	0.24, 1.65	0.35	0.51	0.22, 1.13	0.10
**Patient's income**						
Without income	—	—				
Less than 1SM	0.80	0.28, 2.27	0.68			
More than 1SM	0.81	0.21, 3.05	0.75			
**Previous TB treatment**						
No	—	—		—	—	
Yes	0.37	0.12, 1.13	0.085	0.40	0.15, 1.06	0.071
**BMI**						
Healthy Weight	—	—				
Underweight	0.56	0.19, 1.62	0.29			
Overweight	0.71	0.20, 2.39	0.58			

	Model 1 (p-value<0.2)			Final Model		
	OR[1]	95% CI[1]	p-value	OR[1]	95% CI[1]	p-value
Obesity	0.19	0.01, 1.36	0.15			
**Hypertension**						
Presence	—	—		—	—	
Absence	2.14	0.52, 9.77	0.30	3.18	0.95, 12.5	0.075
**Smoking**						
Does not require intervention	—	—				
Needs a brief intervention	0.63	0.22, 1.71	0.37			
Need more intensive treatment	5.84	0.51, 67.0	0.16			
**Drugs**						
Does not require intervention	—	—				
Needs a brief intervention	0.85	0.22, 3.32	0.82			
Need more intensive treatment	0.39	0.02, 9.11	0.56			
**Cavitation on chest radiograph**						
Absence	—	—		—	—	
Presence	15.8	5.86, 48.1	<0.001	13.7	5.93, 34.5	<0.001
**Dysglycemia**						
Absent	—	—				
Prediabetes e Diabetes	2.06	0.81, 5.39	0.13			
**Anemia**						
Absent	—	—				
Present	0.76	0.24, 2.28	0.63			
**Neutrophils**						
Normal/Low	—	—		—	—	
High	2.36	0.78, 7.43	0.13	2.15	0.81, 5.96	0.13
**Lymphocytes**						
Normal	—	—				
Low	1.72	0.57, 5.32	0.34			
High	0.60	0.04, 5.55	0.68			
**Ferritin**						
Normal	—	—				
High	0.71	0.24, 2.11	0.54			
**Albumin**						
Normal	—	—				
Low	2.21	0.72, 6.81	0.16			
**Alkaline phosphatase**						
Normal	—	—		—	—	
High	2.97	1.14, 8.11	0.028	3.89	1.68, 9.47	0.002
**C-reactive protein (mg/L)**						

	Model 1 (p-value<0.2)			Final Model		
	**OR[1]**	**95% CI[1]**	**p-value**	**OR[1]**	**95% CI[1]**	**p-value**
<6.0	—	—		—	—	
6.0 a 10.0	0.56	0.12, 2.30	0.44	0.59	0.16, 1.99	0.40
> 10.0	4.07	1.18, 14.7	0.028	5.60	2.23, 14.7	<0.001
**Mini Plus**						
Improbable depression diagnosis	—	—		—	—	
Current major depressive episode	0.27	0.08, 0.87	0.030	0.33	0.11, 0.91	0.036
Past major depressive episode	2.50	0.46, 14.8	0.30	2.51	0.56, 12.8	0.24

[1] OR = Odds Ratio, CI = Confidence Interval.
